# Human Disease-Drug Network Based on Genomic Expression Profiles

**DOI:** 10.1371/journal.pone.0006536

**Published:** 2009-08-06

**Authors:** Guanghui Hu, Pankaj Agarwal

**Affiliations:** Computational Biology, GlaxoSmithKline, King of Prussia, Pennsylvania, United States of America; Georgia Institute of Technology, United States of America

## Abstract

**Background:**

Drug repositioning offers the possibility of faster development times and reduced risks in drug discovery. With the rapid development of high-throughput technologies and ever-increasing accumulation of whole genome-level datasets, an increasing number of diseases and drugs can be comprehensively characterized by the changes they induce in gene expression, protein, metabolites and phenotypes.

**Methodology/Principal Findings:**

We performed a systematic, large-scale analysis of genomic expression profiles of human diseases and drugs to create a disease-drug network. A network of 170,027 significant interactions was extracted from the ∼24.5 million comparisons between ∼7,000 publicly available transcriptomic profiles. The network includes 645 disease-disease, 5,008 disease-drug, and 164,374 drug-drug relationships. At least 60% of the disease-disease pairs were in the same disease area as determined by the Medical Subject Headings (MeSH) disease classification tree. The remaining can drive a molecular level nosology by discovering relationships between seemingly unrelated diseases, such as a connection between bipolar disorder and hereditary spastic paraplegia, and a connection between actinic keratosis and cancer. Among the 5,008 disease-drug links, connections with negative scores suggest new indications for existing drugs, such as the use of some antimalaria drugs for Crohn's disease, and a variety of existing drugs for Huntington's disease; while the positive scoring connections can aid in drug side effect identification, such as tamoxifen's undesired carcinogenic property. From the ∼37K drug-drug relationships, we discover relationships that aid in target and pathway deconvolution, such as 1) KCNMA1 as a potential molecular target of lobeline, and 2) both apoptotic DNA fragmentation and G2/M DNA damage checkpoint regulation as potential pathway targets of daunorubicin.

**Conclusions/Significance:**

We have automatically generated thousands of disease and drug expression profiles using GEO datasets, and constructed a large scale disease-drug network for effective and efficient drug repositioning as well as drug target/pathway identification.

## Introduction

Traditionally, human diseases are classified according to the observational correlation between pathological analysis and clinical syndromes via a reductionist approach [1], [2]. Although serving the clinicians fairly well to date, this classification suffers from a lack of sensitivity to detect diseases before the appearance of symptoms and ambiguity in disease diagnosis [1], [3]. In a similar vein, the traditional view of drug action on disease as a “key” fitting into the “lock” is certainly over-simplified and has been challenged by a growing body of evidence showing that there are many keys for each lock and a single key can fit multiple locks [4], [5]. The existence of unwanted drug side effects and high rate of safety-related drug failures also suggests that the current efforts of identifying highly selective compounds based on limited comparative assays may be ineffective. As argued by Loscalzo et al., the above shortcomings could be alleviated to a great extent by a “network” approach that both appreciates the use as well as the limits of reductionism and incorporates the tenets of the non-reductionist approach of complex systems analysis [1], [5]. The latter component becomes increasingly feasible in the post-genomic era because of the advent of high-throughput technologies (such as genomics, transcriptomics, proteomics, metabolomics, phenomics, etc.). This enables an automated, somewhat comprehensive monitoring of the changes of various molecular components associated with different disease states and drug treatments, therefore enables the characterization of disease and drug effects, and an elucidation of their relationships at a molecular systems level [6]–[12].

Here we generate a large-scale disease-disease, drug-drug and disease-drug network by directly matching their molecular profiles; in particular, their transcriptomic profiles thanks to the accumulation of whole-genome gene expression data available in the public domain. The main assumption of our approach is that gene expression profiles of many (but not all) diseases and drugs can characterize to some extent the effects of disease and drugs; therefore, these diseases and drugs can be related based on the similarity/dissimilarity of their induced expression profiles. This assumption, though not without caveats and limitations, has been generally validated by numerous studies, including the recent seminal work on the Connectivity Map [13]–[16].

We also analyzed the transcriptomic effects of other agents (such as tool compounds and infections) besides FDA-approved drugs. For convenience, we collectively call any such agent that causes potential perturbation in a biological sample a drug, and we use disease-drug network as a general term referring to an interconnected network containing all three types of links, namely disease-disease, disease-drug, and drug-drug links. Our analysis showed that the derived “disease-drug network” may not only provide insights into how we can improve drug discovery for complex diseases, but also provide a “rational” way for systematic drug repositioning, target and pathway deconvolution, and identification of potential side effects for closer monitoring.

## Results

### Generating human disease and drug genomic profiles using GEO microarray datasets

We used human GEO datasets to generate human disease and drug genomic profiles. A GEO DataSet (GDS) represents a collection of biologically- and statistically-comparable samples processed using the same platform [17]. An automatic process was used to extract every subgroup of samples, and perform pair-wise comparison between any two biologically-comparable subgroups [18]. For more reliable results, we excluded any subgroup without replication from the comparisons, and we applied the cyber-T test (instead of a standard t-test) for comparative analysis to appropriately account for the small sample size issue common in GEO data sets [19]. In total, 4,936 comparative analyses were carried out using GEO DataSets. This includes 395 comparisons between pairs of disease states, or diseases versus controls, and also coincidently includes 395 comparisons between pairs of drugs or drugs versus controls. Only these combined 790 disease or drug related profiles (as in Supplementary Table S1 online) were used for further analysis. The remaining 4,146 comparisons were from differences in age, cell line, cell type, development stage, dose, genotype/variation, protocol, species, temperature, time, tissue and others, and are excluded from this study.

### Generating human disease–drug networks using GEO microarray datasets

To establish the links between different diseases and drugs, we applied two different methods to calculate the “similarity” between any two of the 790 genomic profiles obtained above. The first method, based on the concept of “correlation”, measures the “profile-profile” similarity by calculating the Pearson correlation of the cyber-T t-statistic values from two profiles. The second method, based on the concept of “enrichment”, measures the “signature-profile” similarity by first generating a signature (a short list of top changed genes) from one profile, then applying a nonparametric technique to assess the non-random distribution of these signature genes in another ranked profile, as previously described in the Connectivity Map (CMap) analysis [16]. This enrichment-based method is critical for expanding the human disease-drug network to data sources (such as curated disease gene sets and many other genomic profiles) where whole genome expression is not available.

In the correlation-based similarity matching, we excluded the genes which were not meaningfully changed (i.e. P≥0.05 or fold change<1.2) in either profile from the calculation. With 790 profiles, we calculated the symmetric correlation for all 311,655 unique pairs. In the following analyses, we focused on a relatively small fraction of these connections that passed a stringent significance criteria based on false discovery rate corrected p-value and number of changed genes (see methods for details). We also limited the set to diseases/drugs in comparison with control (instead of other diseases/drugs) because this subset is presumably more interesting and also easier to interpret. In addition, we excluded those correlating identical or similar effects as well as redundant correlations (e.g. two connections that both relate obesity to type 2 diabetes) by only choosing the ones with higher correlation coefficients. This process generated a total of 898 significant and interesting links (222 disease-disease, 347 drug-drug, and 329 disease-drug) between 149 nodes (with 74 diseases and 75 drugs) (Supplementary Table S2 online). To assess the reliability of these connections, we mapped the connected diseases onto Medical Subject Headings (MeSH) terms. Of the 145 disease-disease links (where each of the pair could be mapped to a MeSH term) with positive correlation, 108 (∼75%) shared at least a common disease area (Table 1 and Supplementary Table S3 online). For example, Ulcerative Colitis and Crohn's disease (with correlation coefficient of 0.86) are both in the Digestive System diseases section of the MeSH tree. Of the remaining 25% disease pairs not located in the same branch of MeSH tree, many of them may still be related biologically. For example, endometriosis has been connected to several type of cancers, not surprisingly, as they are both characterized by cell invasion and unrestrained growth [20], however, they are not explicitly in the same disease area according to MeSH. Moreover, it has been suggested that women suffering from endometriosis are more susceptible to some forms of cancer including ovarian, endocrine, brain and breast cancer [21], [22].

**Table 1:** A manual selection of disease connections and their mapping on MeSH disease classification.

Profile1 or Signature*	Profile2*	MeSH term for profile1/signature	MeSH term for profile2	Level of matched disease in MeSH tree#	Enrichment score (correlation coefficient)
GDS1956.0.3	GDS1956.0.6	Amyotrophic Lateral Sclerosis	Muscular Dystrophy, Emery-Dreifuss	2	1.29
GDS2118.0.1	GDS2118.0.2	Anemia, Refractory	Anemia, Sideroblastic	4	1.58
GDS2118.0.3	GDS2397.0.1	Anemia, Refractory, with Excess of Blasts	Myelofibrosis	3	(0.58)
GDS1321.0.1	GDS1321.0.2	Barrett Esophagus	Adenocarcinoma	0	1.59
GDS2190.0.1	GDS810.0.2	Bipolar Disorder	Alzheimer Disease	1	1.06
GDS2250.0.3	GDS2418.0.1	Carcinoma, Basal Cell	Cervical Intraepithelial Neoplasia	4	1.2
GDS651.0.1	GDS651.0.2	Cardiomyopathy, Dilated	Cardiomyopathy, Restrictive	3	1.42
GDS1989.1.7.0	GDS2418.0.1	Cervical Intraepithelial Neoplasia	Lymphatic Metastasis	1	1.01
GDS1615.0.1	GDS1615.0.2	Colitis, Ulcerative	Crohn Disease	4	(0.86)
GDS2200.0.1	GDS2200.0.2	Keratosis	Carcinoma, Squamous Cell	0	1.5
GDS1989.1.6.0	GDS1989.1.7.0	Lymphatic Metastasis	Melanoma	1	1.49
GDS1989.1.2.0	GDS1989.1.7.0	Lymphatic Metastasis	Nevus	1	1.17
GDS2643.0.1.5	GDS2643.0.3.5	Multiple Myeloma	Waldenstrom Macroglobulinemia	4	1.08
GDS1956.0.2	GDS1956.0.8	Muscular Dystrophy, Duchenne	Dermatomyositis	3	(0.73)
GDS1956.0.6	GDS1956.0.7	Muscular Dystrophy, Emery-Dreifuss	Muscular Dystrophies	5	(0.53)
GDS1956.0.1	GDS1956.0.5	Myopathy, Central Core	Muscular Dystrophy, Facioscapulohumeral	3	(0.57)
GDS1375.0.1	GDS1375.0.2	Nevus	Melanoma	3	1.5
GDS1746.2.3.0	GDS1746.2.4.0	Prostatic Hyperplasia	Prostatic Neoplasms	3	1.09
GDS1439.0.1	GDS1439.0.2	Prostatic Neoplasms	Neoplasm Metastasis	1	1.09
GDS1282.0.1	GDS1282.0.2	Wilms Tumor	Sarcoma, Clear Cell	2	1.1

Results from both the enrichment score and correlation coefficient method are included in this table. Numbers within parenthesis are correlation coefficients.

* The “names” of profiles or signatures refer to GEO datasets from which profiles or signatures are derived.

# The level number refers to the level that the two connected disease/drug entities are co-located. Level 0 indicates the two diseases are in different disease areas according to MeSH.

In the enrichment-based analysis, we ranked the genes in a profile primarily based on their signed fold changes (i.e. from maximal positive to maximal negative), but with consideration of their associated cyber-T significance P values because both magnitude and significance are important to quantify a differential expression. Indeed, it was reported that gene selections based on fold change in combination with a “generous” P value cut-off (P<0.05) were more reliable (and more consistent with the results from similar studies) than those simply based on P value or fold change alone [23]. For each of 790 profiles, we generated a corresponding signature by extracting the 200 most changed genes (i.e. 100 up-regulated plus 100 down-regulated genes excluding the “hypothetical genes”). We then calculated a total of 623,310 (790×789) enrichment scores for every profile-signature pair (except matching the profile to its own signature), from which we obtained 2,882 non-redundant connections with P<0.05 (equivalent to |enrichment scores|>0.45, Supplementary Table S4 online). The MeSH disease tree was also used to assess the reliability of these relationships. We found that 350 of the 585 (60%) disease links (P<0.05) belong to same disease area (Table 1 and Supplementary Table S5 online), for example, Nevus and Melanoma (with enrichment score of 1.5) are both part of the Neoplasm MeSH tree. Again, some of the significantly connected diseases that are not located in the same branch of MeSH tree may still be biologically related.

Comparison of the two networks constructed from the selected top connections by two different methods showed a statistically significant overlap. For examples, among the top 898 correlation-derived links, 336 (vs. 4.1 by chance) of them also covered by the top 2,882 (P<0.05) enrichment-derived links. Because the results from both methods are fairly reliable as shown by good MeSH validation rates, the relatively low overlap suggests that both methods have relatively low recall or sensitivity. This is consistent with the different designs of the two techniques with the enrichment method relying more on the “local” similarity, while the correlation method depends on the “global” similarity. Therefore, it is beneficial to combine the results to increase the sensitivity in identifying interesting relationships.

**Figure 1:**
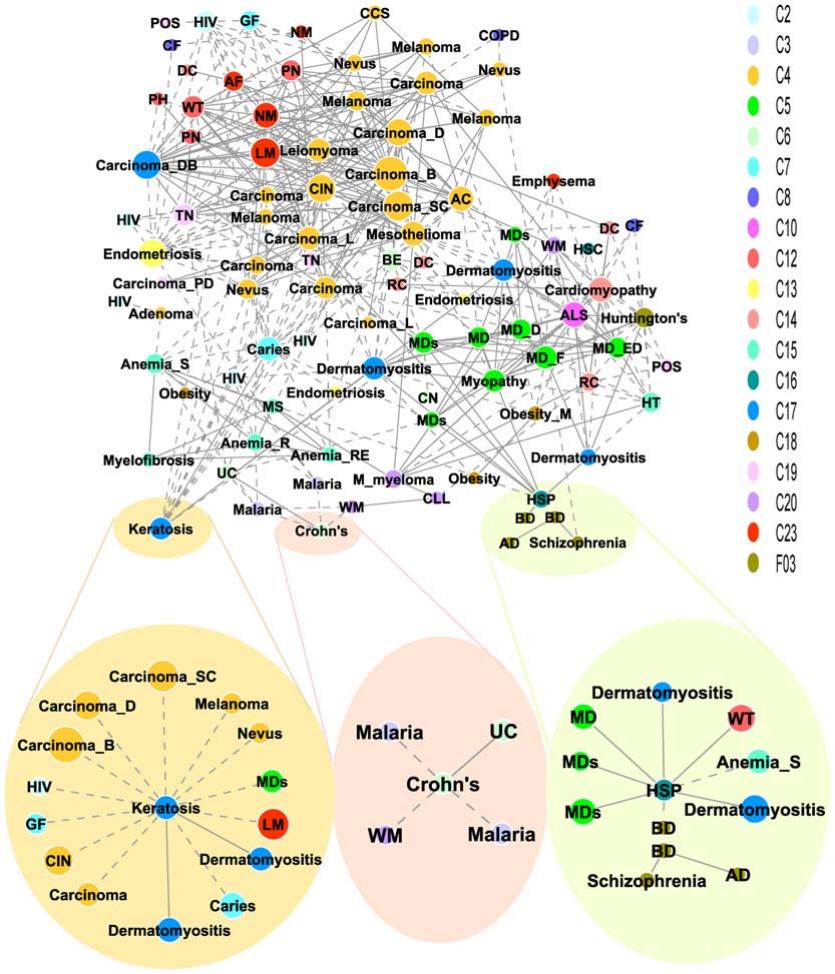
Human disease network. In the disease network, each node corresponds to a disease colored according to their MeSH disease category as denoted by the MeSH Tree Number as shown on the right panel. The size of each node is proportional to the number of diseases connecting to it. A solid line links two diseases from same MeSH disease category while the dot line links two diseases from different MeSH disease category. Multiple nodes may represent the same disease, but they are from different studies or conditions, e.g. there are two bipolar disorder nodes, whose profiles are derived from studies using two different tissues (dorsolateral prefrontal cortex tissue and orbitofrontal cortex tissue). Disease abbreviations used in this figure include: C02, virus disease; C03, parasitic diseases; C04, neoplasms; C05, musculoskeletal diseases; C06, digestive system diseases; C07, stomatognathic diseases; C08, respiratory tract diseases; C10, nervous system diseases; C12, male urogenital diseases; C13, female urogenital diseases and pregnancy complications; C14, cardiovascular diseases; C15, hemic and lymphatic diseases; C16, congenital hereditary neonatal diseases and abnormalities; C17, skin and connective tissue diseases; C18, nutritional and metabolic diseases; C19, endocrine system diseases; C20, immune system diseases; C23, pathological conditions signs and symptoms; F03, mental disorders; AC, adenocarcinoma; AD, Alzheimer disease; AF, atrial fibrillation; ALS, amyotrophic lateral sclerosis; Anemia_R, refractory anemia; Anemia_RE, refractory anemia with excess of blasts; Anemia_S, sideroblastic anemia; BD, bipolar disorder; BE, Barrett esophagus; Carcinoma_B, basal cell carcinoma; Carcinoma_D, ductal carcinoma; Carcinoma_DB, breast ductal carcinoma; Carcinoma_L, lobular carcinoma; Carcinoma_PD, pancreatic ductal carcinoma; Carcinoma_SC, squamous cell carcinoma; CCS, clear cell sarcoma; CF cystic fibrosis; CIN, cervical intraepithelial neoplasia; CLL, chromic lymphocytic leukemia; CN, colorectal neoplasms; COPD, chronic obstructive pulmonary disease; DC, dilated cardiomyopathy; GF, gingival fibromatosis; HSC, hemoglobin sickle cell disease; HSP, hereditary spastic paraplegia; HT, hemorrhagic thrombocythemia; LM, lymphatic metastasis; M_myeloma, multiple myoloma; MD, muscular dystrophy; MD_D, Duchenne muscular dystrophy; MD_ED, Emery-Dreifuss muscular dystrophy; MD_F, Facioscapulohumeral muscular dystrophy; MDs, muscular diseases; MS, myelodysplastic syndromes; NM, neoplasm metastasis; Obesity_M, morbid obesity; PH, prostatic hyperplasia; POS polycystic ovary syndrome; RC, restrictive cardiomyopathy; TN, thyroid neoplasms; UC, ulcerative colitis; WM, waldenstrom macroglobulinemia; WT, Wilms tumor.

The connected diseases that are located in different branches of MeSH tree are particularly interesting, because they provide potentially novel disease relationships that primarily rely on genomic expression profile similarity instead of phenotypic classification. As an example, Bipolar disorder is linked to Alzheimer's disease and Schizophrenia; all three of which are Mental disorders (Figure 1). But it is also linked to Hereditary Spastic Paraplegia (HSP) which is not a Mental Disorder. HSP is a group of inherited disorders characterized by progressive weakness and stiffness of the legs, and therefore is regarded as a neuromuscular disease[24]. Indeed, our disease network also links HSP to a number of muscular diseases such as Dermatomyositis and Muscular Dystrophies (Table 1 and Supplementary Table S6 online). The novel gene expression connection between HSP and Bipolar disorder indicates that they may share some common underlying molecular mechanism. Interestingly, there are some clinical observations that complicated forms of HSP can be accompanied by neurological symptoms including Dementia and Mental retardation [25].

Another example, an Actinic keratosis, also known as a Solar keratosis, is a small, rough spot occurring on skin that has been chronically exposed to the sun [26]. Our disease network links it to a number of cancers (including squamous cell carcinoma and melanoma) in addition to some other benign skin conditions such as Nevus (Figure 1), which provides molecular level support for the clinical warning that Actinic keratoses are precancerous [27].

Besides providing a new way to redefine human diseases and gain a broader understanding of disease mechanism, the genomic profile-based disease relationship can also help us to find potential new indications of existing drugs. The disease sub-network shows that Crohn's disease (a form of inflammatory bowel disease [28]) is linked to the closely related ulcerative colitis as well as some other immune/inflammatory diseases (such as dermatomyositis). More interestingly, we find that Crohn's disease is also linked to malaria (Figure 1). This seemingly surprising connection is supported by emerging evidence that Crohn's disease has potential infectious causes [29], [30]. In fact, it has been proposed that some antimalarial drugs might be also effective against Crohn's disease [31]. This was based on the observation that military duty in Vietnam exerted a protective influence against the development of Crohn's disease, and most American soldiers, while stationed in Vietnam, were prescribed malaria chemoprophylaxis.

### Expanding human disease – drug network

We expanded the disease-drug network by including the 6,100 reference gene-expression profiles from the CMap project [16]. This expanded network contains a total of 170,027 with P<0.05, including 645 disease-disease, 5,008 disease-drug, and 164,374 drug-drug connections. Like many empirically observed biological networks such as the protein-protein interaction network [32]–[34], the disease-drug network is also a scale-free network whose degree distribution follows a power law (data not shown), that is, most nodes connect to only a few other nodes whereas a few nodes act as hubs with a large number of links. The most connected drug hub is Trichostatin A, an organic compound that serves as an antifungal antibiotic and selectively inhibits the class I and II mammalian histone deacetylases [35]. It links to hundreds of other agents and a few diseases, partially because of its large effects on transcription. The most connected disease hubs include many types of cancers, some inflammatory diseases, Neisseria meningitides, Huntington's and Cardiomyopathy, all with at least several dozen links to drugs and other diseases. The prominence of cancers and inflammatory disorders among the most connected diseases is partially because they have many subtypes or related conditions sharing same biological dysfunctions. For examples, many cancers involve common tumor activators (such as Ras and Myc) or tumor suppressors (such as p53 and PTEN) [36], while most inflammatory diseases are associated with the changes of cytokines and chemokines [37]. This expanded human disease-drug network may be used as a starting point in human disease reclassification, target and pathway deconvolution, drug repositioning and elucidating potential side effects, some of which are described in more details in the following sections.

### Disease-drug connections: drug repositioning and side effects

**Figure 2:**
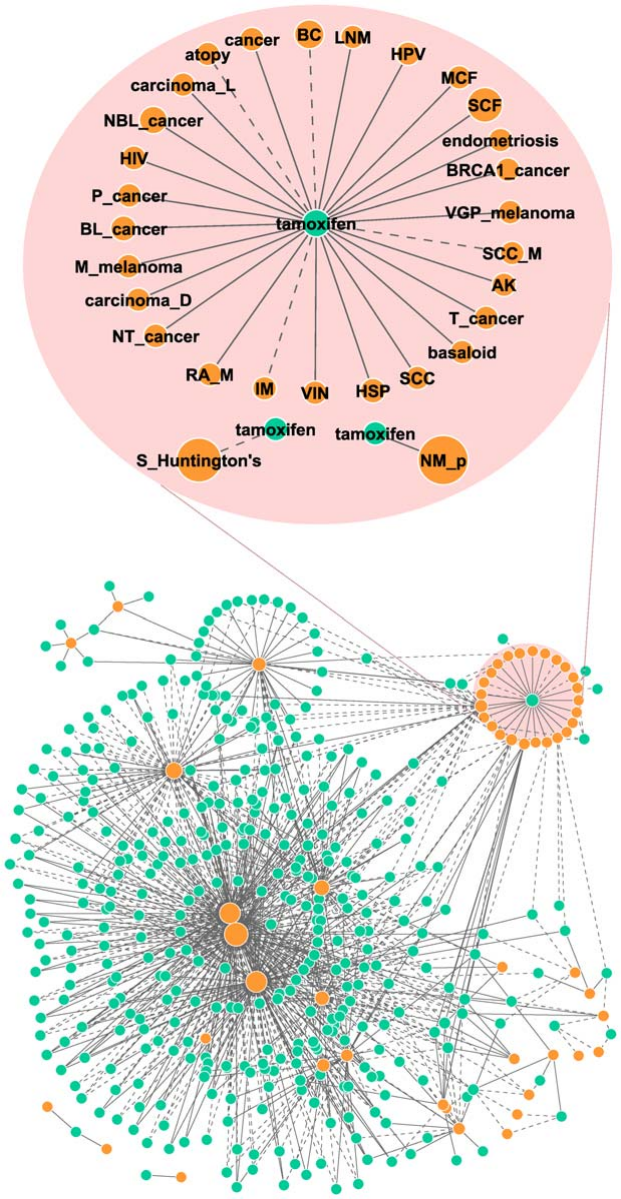
Disease-drug network. This disease-drug network contains a total of 49 diseases in dark cyan nodes, 213 drugs in gold, and 906 connections. The size of the nodes is proportional to the number of links. Positive matches are shown by solid lines and negative relationships by dotted lines. Multiple nodes with the same descriptive name exist because the corresponding profiles were generated under different conditions or studies (refer to Supplementary Table S7 online for details). In addition to the abbreviations listed in the Figure 1 legend, other abbreviations used in this figure include: AK, actinic keratosis; BC, breast cancer; BL_cancer, basal_like cancer; BRCA1_cancer, BRAC1-associated cancer; HPV, human papillomavirus; IM, idiopathic myelofibrosis; LNM, lymph node metastasis; MCF, mild cystic fibrosis; NBL_cancer, non-basal-like cancer; NT_cancer, non-tumorigenic cancer cell; P_cancer, metastatic prostate cancer; RA_M, rheumatoid arthritis on methotrexate; SCC, squamous cell carcinoma; SCC_M, squamous cell carcinomas (lymph node metastasis); SCF, severe cystic fibrosis; T_cancer, tumorigenic cancer cell; VGP_melanoma, vertical growth phase melanoma; VIN, vulvar intraepithelial neoplasia.

We extracted a disease-drug sub-network consisting of unique and significant connections (i.e. P<0.05 for enrichment-derived links; FDR-corrected P<1e-10, and |r|>0.3 for correlation-derived links), with the drugs limited to those annotated by DrugBank as of July 25, 2008 [38]. This sub-network, containing a total of 906 non-redundant disease-drug links, 49 diseases and 213 drugs (Figure 2, Table 2 and Supplementary Table S7 online), allows us to generate hypotheses on potential drug side effects and drug repositioning. For example, the network suggests that drugs for the treatment of Neurological disorders, Hypertension/Heart diseases, Cancer, AIDs, Migraine Headaches, and Inflammation may also help in Huntington's disease (Supplementary Table S8 online). Huntington's disease is a neurodegenerative disease characterized by the build up of malformed proteins in brain cells, mainly in the basal ganglia and the cerebral cortex [39]. It has previously been shown that stimulating autophagy in the cells can be an effective way of preventing the build up of malformed proteins. A number of drugs for the treatment of Migraine and Hypertension have been able to stimulate autophagy in fruit flies and zebrafish [40], and therefore are potentially drug candidates for Huntington's. Rapamycin, an immunosuppressant used to lower the body's natural immunity in patients who receive kidney transplants, is a promising drug for Huntington's, also likely via its autophagy-inducing function [41]. Another promising area of research is certain cancer and AIDS drugs. It has been shown that some cancer drugs in combination with AIDs drugs halt the progress of Huntington's in fruit flies [42], [43]. We also found some existing drugs for Diabetes, Glaucoma and Gout connected with negative scores to Huntington's (Supplementary Table S8 online), suggesting that they may be candidate drugs for Huntington's as well.

**Table 2:** A manual selection of a few disease-drug connections.

Drug	Disease	Enrichment score (correlation coefficient)
Tamoxifen	Atopy	(−0.58)
Tamoxifen	Basal-like cancer	0.95
Captopril	Benign nevi	−0.83
Etoposide	Breast cancer (adenovirus carrying estrogen receptor beta)	0.82
Tamoxifen	Breast cancer (adenovirus carrying estrogen receptor beta)	−1.41
Tamoxifen	Endometriosis	0.86
Apomorphine	Huntington's disease (symptomatic)	−0.82
Dacarbazine	Huntington's disease (symptomatic)	−0.84
Ethosuximide	Huntington's disease (symptomatic)	−0.85
Haloperidol	Huntington's disease (symptomatic)	0.86
Remoxipride	Huntington's disease (symptomatic)	−0.83
Bumetanide	Neisseria meningitidis (delta pilD mutant)	0.82
Fenoprofen	Neisseria meningitidis (delta pilD mutant)	−0.81
Gliclazide	Neisseria meningitidis (delta pilD mutant)	−0.82
Haloperidol	Neisseria meningitidis (delta pilD mutant)	0.83
Levocabastine	Neisseria meningitidis (delta pilD mutant)	0.84
Metolazone	Neisseria meningitidis (delta pilD mutant)	−0.81
Verapamil	Neisseria meningitidis (delta pilD mutant)	0.83
Tamoxifen	Non-basal-like cancer	−0.7
Diltiazem	Non-ischemic cardiomyopathy	−0.88
Diphenhydramine	Non-ischemic cardiomyopathy	0.84
Ethosuximide	Non-ischemic cardiomyopathy	−0.86
Fenoprofen	Non-ischemic cardiomyopathy	−0.87
Glipizide	Non-ischemic cardiomyopathy	−0.81
Paclitaxel	Non-ischemic cardiomyopathy	−0.82
Valproic acid	Polycystic ovary syndrome	0.92
Tamoxifen	Prostate cancer (metastatic)	0.95

Results from both the enrichment score and correlation coefficient method are included in this table. Numbers within parenthesis are correlation coefficients.

Another example, the anti-breast cancer drug Tamoxifen is linked with a negative score (in the network) to Atopy, Huntington's disease, and Idiopathic myelofibrosis besides the expected Breast cancer (Figure 2). This suggests that in addition to acting as an anti-breast agent via antagonizing estrogen receptor, Tamoxifen could also be possibly used as a therapy for other diseases such as Atopy. These hypotheses are aligned with some published studies [44]–[49]. For example, tamoxifen inhibits mast cell secretion in a rat study, probably via PKC [47]. The mast cell's critical role in allergic reactions indicates that this is consistent with the negative connection between them in our results. Interestingly, we find that tamoxifen is “positively” (i.e. similar profile patterns with positive correlations and/or enrichment score) linked to many types of cancers and other disorders (namely, Endometriosis, Cystic fibrosis, HPV positive and early HIV infection) that share common underlying biological processes with cancer (such as cell invasion, uncontrolled growth, and weakened immunity etc.). This suggests that Tamoxifen may have an undesired “carcinogenic” property. Indeed, Tamoxifen causes an increased incidence of Endometrial cancer in human [50] and Liver cancer in rat [51]. When Tamoxifen is administrated to neonatal rats, Uterine adenocarcinomas were induced along with a lower frequency of Squamous cell carcinomas of the vagina/cervix [52].

### Drug-drug connections: target and pathway deconvolution

**Figure 3:**
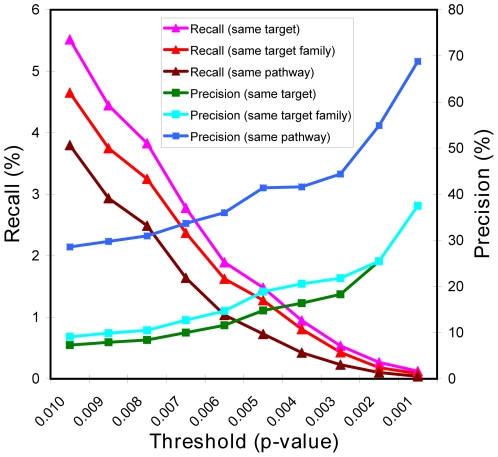
The precision and recall of target and pathway deconvolution. Precision is the fraction of the identified targets or pathways that are correct, calculated as “true positive”/(“true positive” + “false positive”). Recall is the fraction of all true targets or pathways that are successfully identified, calculated as “true positive”/(“true positive” + “false negative”).

Drugs with similar expression profiles may target the same molecules or biological pathways. We used the known drug-target relationships from DrugBank [38] to assess this. The DrugBank includes 1,692 approved/experimental drugs spanning 743 human protein targets. 360 of the 1,692 drugs were also covered by our drug profile data. These 360 drugs had 3,668 connections in the expression network, with an enrichment |score|>0.74 (corresponding to an empirical P value of 0.01), of which 7.3% shared at least one common target (Figure 3 and Supplementary Table S9 online). The actual chance of sharing a target is likely to be higher because only the drug-target information documented in the DrugBank was used, many binding partners for known drugs are not documented by DrugBank or not known yet and were thus counted as false negatives. The precision increases as the enrichment score/significance threshold increases, though the recall decreases as expected (Figure 3 and Supplementary Table S9 online). For example, for the connections with enrichment |score|>1.25 (i.e. P<0.002), about 25% (13 out of 51) of them shared at least one common target, which is more than 6-fold higher than what would be expected by random chance (3.8%). Because proteins from same family often have similar tertiary structures and active sites, if a compound binds a protein target, it will likely have affinity with some of its family members as well. Indeed, the percentage of the connected drugs sharing at least one target from the same protein family is generally higher (Figure 3 and Supplementary Table S9 online), even when we only restricted ourselves to just the obvious family members.

We examined the top 249 connections (with an enrichment score cut-off of 1.25, corresponding to a precision of ∼25%) that linked drugs to those with known targets (Table 3 and Supplementary Table S10 online). For example, our results suggest that the potassium large conductance calcium-activated channel KCNMA1 may be a protein targeted by Lobeline, a natural alkaloid that has been used as a smoking cessation aid and may have application in the treatment of addictions to drugs such as Amphetamines or Cocaine [53]–[55]. The hypothetical link of KCNMA1 with Lobeline appears to be consistent with a recent report demonstrating the role of KCNMA1 in neuronal excitability [56].

**Table 3:** A manual selection of compounds with predicted drug target(s) based on similarity in expression profiles to another drug with known target.

Drug	Predicted Target(s)	Predicted targeting pathways
Ajmaline	KCNMA1 HRH2	GPCRs in the regulation of smooth muscle tone; G-Protein Coupled Receptor Signaling; cAMP-mediated Signaling
Cicloheximide	ATP1A1	Leptin signaling via PI3K-dependent pathway
Lobeline	KCNMA1	GPCRs in the regulation of smooth muscle tone
Quercetin	NR3C2 FKBP1A	Glucocorticoid Receptor Signaling; IL-4 Signaling; Neutrophil and Its Surface Molecules; mTOR Signaling Pathway
Salbutamol	DRD2	Regulation of cell cycle progression by Plk3; Dopamine Receptor Signaling; G-Protein Coupled Receptor Signaling; cAMP-mediated Signaling
Strophanthidin	ATP1A1	Leptin signaling via PI3K-dependent pathway
Daunorubicin	TOP2A	Apoptotic DNA fragmentation and tissue homeostasis; Cell Cycle: G2/M DNA Damage Checkpoint Regulation

The last column is the list of pathways that the target of the known drug participates. Thus, the hypothesis is that the shown drug may act either through the predicted target or at least through the shown pathway.

We next calculated the percentages of the connected drugs perturbing at least one common well-defined biological pathway at specified thresholds of enrichment score. We used pathways from three manually-curated data sources: Biocarta (208 pathways, 1,321 unique genes) (http://www.biocarta.com), Ingenuity (166 pathways, 4,085 unique genes) (http://www.ingenuity.com) and GeneGo (515 pathways, 2,685 unique genes) (http://www.genego.com). Because there are substantial overlaps among them, the total number of unique genes covered by the 3 pathway data sources is only 5,166. 1,048 of 3,668 (∼28.6%) of the related drug pairs (with enrichment |score|>0.74 corresponding to an empirical P<0.01) targeted the same pathway (Figure 3 and Supplementary Table S9 online). Moreover, for connections with enrichment |score|>1.25 (i.e. P<0.002), more than half (28 out of 51, vs. 21.3% by random chance) of connected targets participate in at least one common pathway. At this threshold (and an expected precision of 50%), 249 connections were generated, from which we identified potential pathways targeted by 116 unique drugs (Table 3 and Supplementary Table S11 online). As an example, two Anthracycline chemotherapy drugs: Daunorubicin and Doxorubicin were found to be significantly connected due to a high similarity of their gene expression profiles. Doxorubicin targets topoisomerase II alpha (TOP2A), which is involved in the biological pathways of apoptotic DNA fragmentation and G2/M DNA damage checkpoint regulation in cell cycle [57]. This suggests that Daunorubicin may exert its anti-cancer function by perturbing the same two pathways. This hypothesis is consistent with the general thought that the cytotoxicity mediated by Daunorubicin is the result of drug-induced damage to DNA [58].

## Discussion

Disease-drug relationships are of great interest because such knowledge can not only significantly enhance our understanding of disease mechanisms, but also accelerate many aspects of drug discovery. We took advantage of an ever-increasing accumulation of whole genome gene expression data to generate a large-scale disease-drug network. This network provides a valuable resource to revisit disease classification (nosology), to reposition therapeutic agents, identify potential drug side effects and deconvolute drug targets or pathways in a cost-effective way. It is worth noting that this network suggests many testable hypotheses with potentially fairly good chances of success, though the actual success rate can only be determined by experimental validation. A contribution of this work is the establishment of an automatic process that allows us to efficiently scale-up and update our disease-drug network when more gene expression data are generated, deposited and annotated as GDS datasets in GEO.

For the significantly-associated disease pairs, the majority (∼70%) of them were positively connected, while the rest (∼30%) were negatively linked (Supplementary Table S12 online). Although it is possible that the two diseases in some of these negatively connected pairs are mutually exclusive (that is to say, if a person has one disease, it will protect the person from having the other disease), we found that most of these pairs merely reflect the existence of some inversely regulated biological processes. For example, a Nevus is a benign overgrowth of skin pigment forming cells called melanocytes on the skin surface, present at birth or appearing early in life [59]. It was found to be negatively linked to some “cancer-like” conditions (such as Adenocarcinoma, Colorectal neoplasms, and Barrett's esophagus) and Muscular diseases (including several types of Muscular dystrophy, Myopathy and Dermatomyositis etc.). The dissimilarity of nevus with “cancer-like” conditions may be because it is benign in contrast of cancerous, while the inverse relationship with muscular weakness/wasting might be due to its characteristic cell overgrowth.

Although our approach has a number of advantages, such as scalability, efficiency and reliability, it is not without shortcomings. One major issue is the high false negative rate, for example, it is not uncommon to find that two similar or “identical” diseases have a very low correlation/enrichment score. That is possibly because the gene expression profiles under comparison were generated under different conditions, such as different tissue samples, cell lines/types, treatment doses and time durations, disease/development stages, ages, genotypes/variations, and experiment protocols etc. The “recovery” rate (i.e. Recall) is expected to increase as more data sets are generated under similar conditions. Connectivity Map already goes a long way towards at least providing many data sets for drugs. What is needed is a similar comprehensive effort for diseases. Another problem of our approach is that it relies on gene expression data alone; therefore it may fail to match disease and drug effects that are not manifested at the gene expression level. However, because the methodology *per se* is general, one approach to this issue is to apply a similar method to other types of Omic data (such as proteomic and metabolomic data) when similar repositories become available.

Integrating other results obtained via conceptually different approaches may also improve the reliability and sensitivity. For examples, we can add links (or modify scores) between two diseases that share at least one gene whose mutations are associated with both diseases [7]. Moreover, diseases may be related using statistical analysis of patient records [10], quantitative measurements of the phenotypic overlap of Online Mendelian Inheritance in Man (OMIM) records [11], and annotative concepts [6], and metabolic diseases may be connected via metabolites and common reactions [9]. For the drug-drug relationship and target deconvolution, we can integrate the results derived from assessing drug side-effect and chemical similarity, target sequence similarity and drug-target network [4], [12], [60]. The disease-drug association results could also be improved by data mining of medical records and biomedical literature [61], [62].

## Methods

### GEO

GEO datasets (GDS) are reassembled by GEO staff from user deposited gene expression data [17]. We downloaded the GEO datasets from ftp://ftp.ncbi.nih.gov/pub/geo/DATA/SOFT/GDS. As of March 17, 2008, the database contains 2,085 sets of GDS entries, including 306 datasets generated from human studies using GPL96 (Affymetrix U133A chip) or GPL570 (U133plus2) platform (Supplementary Table S13 online). All of our analyses were limited to genes commonly covered by both U133A and U133plus2 chips. In addition, we also queried the GEO website (http://www.ncbi.nlm.nih.gov/geo/) for “GPL96” and “GPL570” to obtain the annotation summary for each of the datasets.

### Identification of single most appropriate Affymetrix probe set for gene

In Affymetrix U133 microarray chips, many genes are represented by multiple probe sets. To avoid correlation/scoring biases brought by such over-representation during similarity-matching, we identified and used a single probe set as the sole representative for each gene as follows. The 11 individual probes (25mers) from each probeset were blasted against the NCBI RefSeq database. The distance to the 3′end for each probeset was calculated as the average distance of 11 individual probes. For those probesets which do not match any RefSeq sequences, they were mapped onto assembled human genome using BLAT: 1) when a probeset is less than 100 bp downstream from a RefSeq, it is defined as “derived from 3′UTR” of that RefSeq sequence; 2) when a probeset is less than 10 kb downstream from a RefSeq, it is defined as “derived from putative 3′UTR” of that RefSeq sequence; 3) when a probeset is located within the coordinates of a RefSeq but does not overlap, its is defined as “derived from potential alternative transcription”; 4) when a probeset is located within the coordinates of a RefSeq but on the opposite strand, its is defined as “derived from potential antisense transcription”. To identify the single most appropriate probe set for each gene, we adopted the following preference order of probe sets derived from: 1) 3′ UTR; 2) coding region with shortest distance to 3′ UTR; 3) putative 3′ UTR; 4) sequence representing potential alternative transcription; 5) sequence representing potential anti-sense transcription; 6) sequence not associated with known genes (in this case, we assigned the probe set ID's as their “gene names”). In the end, we obtained 26,201 probe sets uniquely representing each gene/transcript covered by U133plus2 chip (Supplementary Table S14 online).

### Generating disease/drug profiles from GEO GDS datasets

For each GEO GDS dataset together with its associated annotation summary, a Python program modified from a previous version [18] was used to extract every subgroup of samples with clearly defined conditions, generate pairs between any two biologically comparable subgroups, and perform cyber-T test for each of these pairs. For example, if a dataset has two subset types T and D (T for “time point” and D for “dose of treatment”), and each subset type has two conditions T1/T2 (for two different time points) and D1/D2 (for two different doses), then 4 sample subgroups are generated: T1D1, T1D2, T2D1 and T2D2, and 4 pair-wise comparisons via cyber-T test are performed: T1D1 vs. T2D1 (i.e. T1 vs. T2 at fixed D1), T1D2 vs. T2D2 (i.e. T1 vs. T2 at fixed D2), T1D1 vs. T1D2 (i.e. D1 vs. D2 at fixed T1), and T2D1 vs. T2D2 (i.e. D1 vs. D2 at fixed T2). The possible types of conditions include disease state, agent treatment, time, tissue, infection, age, cell line, cell type, development stage, treatment dose, genotype/variation, growth protocol, protocol, species, specimen, stress, temperature, and others. For more reliable results, we excluded any subgroups without replication from the comparisons. The result was a profile for each disease/drug, containing the fold change, signed cyber-T t-statistic and P value of differential expression for each probe set. These profiles were then filtered to retain the comparative analysis information limited to those most appropriate probe sets identified above. We also replaced the probe sets with their corresponding HUGO gene symbols if they existed.

### Correlation calculation

The signed cyber-T t-statistic values were used to calculate a Pearson correlation [18]. For each pair of profiles, we only included those probe sets that are the most appropriate gene representatives, and they must be “meaningfully” changed (p<0.05, and fold change>1.2) in at least one of the profiles. In addition, the number of “meaningfully” changed genes in each profile must be more than 100. R code was used to calculate the correlation significance P values, and Storey's FDR method was used calculate the false discovery rate q values via the R package “QVALUE” [63]. We chose an extremely conservative FDR cut-off of p<1e-18 in this paper.

### Connectivity Map profile database and process

The Connectivity Map (CMap) is a collection of genome-wide transcriptional expression data from cultured human cells treated with bioactive small molecules [16]. As of July 8, 2008, CMap contains 6,100 expression profiles representing 1,309 compounds. We downloaded the data file “rankMatrix.txt.zip” and its associated annotation file “cmap_instance_02.xls” from the CMap website (http://www.broad.mit.edu/cmap/). We kept only the single most appropriate probe set for each gene, and replaced the probe sets with their corresponding HUGO gene symbols.

### Disease and drug signatures

A signature is a relatively short list of genes associated with disease or drug effects, and can be derived either by manual curation or automated filtering from high-throughput experiments. In this work, signatures are directly derived from disease/drug expression profiles by taking the most changed non-redundant genes. We first removed hypothetical and not-significantly-changed (P≥0.05) genes if the P-value is available; then selected a total of 200 genes with maximal fold changes (100 positive, and 100 negative each). The size of signature (i.e. 200 genes) was chosen primarily based on our experience and testing of the impact of different sizes (50, 100 and 200) on signature-profile matching scores. We found empirically that any size from a few dozens to a few hundreds did not affect the results qualitatively, while signatures with too few or too many genes led to lower sensitivity and specificity in similarity detection.

### Enrichment scores

We first reformatted each disease/drug profile by ranking the probe sets according to their signed fold changes. For those profiles with P values (e.g. those generated from GEO datasets), significantly (p<0.05) and insignificantly (p≥0.05) changed probe sets are ranked separately by their fold changes first, and then merged by inserting the ranked but insignificantly changed probe sets into the +/− fold change boundary of ranked significant ones. We then generated a signature from each profile, and assessed the similarity between the signatures and the profiles by quantitatively measuring the enrichment of signature genes in the top/bottom ranked region of the profiles, similar to as previously described in CMap [16]. We generated a score distribution generated from 1.5 million real data points, showing any |score|>0.74 indicates a p-value of less than 0.01, and |score|>0.45 indicates a p-value of less than 0.05. Random permutation test was also used to assess the significance of enrichment scores. Based on a score distribution generated from 1 million permutations, any non-zero score is statistically significant, therefore potentially interesting.

### MeSH thesaurus and disease mapping

MeSH is the National Library of Medicine's controlled vocabulary thesaurus. It consists of sets of terms naming descriptors in a hierarchical structure that permits searching at various levels of specificity. We downloaded the disease tree file mtree2008.txt from MeSH website (http://www.nlm.nih.gov/mesh/), which, as of June 17, 2008, contains 48,443 subjects and 24,766 unique descriptors grouped in 16 categories, including Disease category, Chemicals and Drugs category, and Pharmacological Actions category etc. Many disease/drug names used in GEO and CMap do not match corresponding MeSH terms. For a subset of 360 selected unique disease/drug terms used in GEO and CMap, only 86 of them can be directly matched to MeSH terms. Of the remaining 274 terms, 120 have also been covered by MeSH but with slightly different names, which we manually corrected. In total, we have 206 matched terms that allowed us to do disease/drug mapping. For matched pairs, Perl scripts and MySQL queries were used to obtain the level of matching in the MeSH hierarchical tree structure. The level indicates the distance from the tree root for the lowest common ancestor of the two connected diseases/drugs. Level 0 indicates that the two diseases/pertubagens belong to different MeSH categories.

### DrugBank database and target deconvolution

The DrugBank database stores drug data with corresponding drug target and treatment indication information [38]. As of July 25, 2008, the database contains nearly 4,800 drug entries, including>1,480 FDA-approved small molecule drugs, 128 FDA-approved biotech drugs, 71 nutraceuticals and more than 3,200 experimental drugs. We selected the drugs which are known to have human target proteins. Perl scripts and MySQL queries were used to match drugs to DrugBank, calculate precision and recall rates, and make prediction of potential targets for drugs whose targets are unknown. To determine whether two proteins are from the same family, we used a simple (and conservative) way by checking whether their HUGO gene symbols only differ at their ending numbers (such as PTGER1 and PTGER2). To determine the expected percentage of two drugs sharing at least one common target by random chance, we generated 10,000 random drug pairs, and assessed how many of them target at least one common molecule according to the DrugBank drug-target information. We also generated all the possible drug pairs (a total of 129,240) and identified all the targets (4,858) and same family targets (7,200) to calculate the fractions as expected rates. Both methods resulted in similar expected percentages.

### Pathway databases and pathway deconvolution

3 curated canonical pathway resources were used in this work: GeneGO (www.genego.com), Ingenuity (www.ingenuity.com) and Biocarta (www.biocarta.com).

### Precision and recall of target and pathway deconvolution

Precision is the fraction of the identified targets or pathways that are correct, calculated as “true positive”/(“true positive” + “false positive”). Recall is the fraction of all true targets or pathways that are successfully identified, calculated as “true positive”/(“true positive” + “false negative”).

## Supporting Information

Table S1 The combined 790 disease or drug related profiles
(0.31 MB TXT)

Table S2 898 significant and interesting disease-drug links
(0.15 MB TXT)

Table S3 Mapping the correlation-derived connected diseases onto MeSH terms
(0.01 MB TXT)

Table S4 2882 non-redundant connections with p<0.05 via enrichment analysis
(0.31 MB TXT)

Table S5 Mapping the enrichment-derived connected diseases onto MeSH
(0.05 MB TXT)

Table S6 Combined disease connections via both enrichment and correlation analysis, and their mapping onto MeSH
(0.03 MB TXT)

Table S7 Extracted disease-drug subnetwork
(0.20 MB XLS)

Table S8 Drugs showing counter-Huntington's disease effects
(0.03 MB DOC)

Table S9 Target and pathway deconvolution
(0.04 MB DOC)

Table S10 The top 249 connections that linked drugs to those with known targets
(0.00 MB TXT)

Table S11 The potential pathways targeted by 116 unique drugs
(0.08 MB TXT)

Table S12 Negatively connected diseases
(0.01 MB TXT)

Table S13 GDS datasets
(0.03 MB TXT)

Table S14 Probe sets uniquely representing each gene/transcript covered by U133plus2 chip
(1.93 MB TXT)
