# Supplementary material for: Human Disease-Drug Network Based on Genomic Expression Profiles
Source: PLoS One. 2009 Aug 6;4(8):e6536. doi: 10.1371/journal.pone.0006536 (PMC2715883; doi:10.1371/journal.pone.0006536)
Supplement: Table S8 — Drugs showing counter-Huntington's disease effects (0.03 MB DOC) [file pone.0006536.s008.doc]

**Supplementary Table. Drugs showing counter-Huntington’s disease effects**

| **Drug Group** | **Drug Names** | **FDA-Approved Indications** |
| --- | --- | --- |
| Neurological disorders | Apomorphine; Arecoline; Baclofen; Bromocriptine; Clomipramine; Dacarbazine;  Ethosuximide; Ethotoin; Fenoprofen; Fluvoxamine; Galantamine; Haloperidol; Imipramine; Memantine; Mitoxantrone; Paroxetine; Procyclidine; Remoxipride; Riluzole; Risperidone; Sulpiride | Alzheimer's; Amyotrophic lateral; Anxiety; Depression; Hodgkin's disease; Multiple sclerosis; Obsessive compulsive disorder; Parkinson's disease; Petit mal epilepsy; Schizophrenia;  Sclerosis; Severe hyperactivity; Seizures |
| Hypertension & heart diseases | Acebutolol; Alprenolol; Amiloride; Betaxolol; Diltiazem; Disopyramide; Dorzolamide; Doxazosin; Felodipine; Hydroflumethiazide; Isradipine;  Labetalol; Methyldopa; Metolazone; Metoprolol; Milrinone; Minoxidil; Nitrendipine; Propafenone; Ramipril; Reserpine; Tolazoline; Torasemide; Trichlormethiazide | Hypertension;  Heart diseases |
| Cancer/AIDS drugs | Dacarbazine; Doxorubicin; Nilutamide; Paclitaxel; Tamoxifen | Cancer of the lung, ovarian, and breast; Kaposi's sarcoma connected to AIDS Melanoma; Prostate cancer; |
| Migraine headaches | Dihydroergotamine; Nicergoline; Propranolol | Migraine headaches |
| Immunosuppressants | Carbinoxamine; Cetirizine; Cyproheptadine; Diclofenac;  Diflunisal; Etodolac;  Fenoprofen; Flunisolide; Gemfibrozil; Hydrocortisone; Levocabastine; Medrysone; Methotrexate; Methylprednisolone; Nabumetone; Oxaprozin; Prednisolone; Rofecoxib; Terbutaline; Theophylline; Triamcinolone | Allergy; Anaphylactic reactions; Asthma; COPD; Inflammation of the pancreas; Inflammatory manifestations; Osteoarthritis; Pain; Psoriasis; Rheumatoid arthritis |
| Diabetes | Acetohexamide; Gliclazide;  Glipizide; Phenformin; Repaglinide | Diabetes |
| Glaucoma | Dorzolamide; Methazolamide; Physostigmine | Glaucoma |
| Gout | Probenecid; Sulfinpyrazone | Gout and high levels of uric acid in the blood |
