# Supplementary material for: Human Disease-Drug Network Based on Genomic Expression Profiles
Source: PLoS One. 2009 Aug 6;4(8):e6536. doi: 10.1371/journal.pone.0006536 (PMC2715883; doi:10.1371/journal.pone.0006536)
Supplement: Table S9 — Target and pathway deconvolution (0.04 MB DOC) [file pone.0006536.s009.doc]

**Table. Target and pathway deconvolution**

| **Cut-off score** | **Empirical**  **p** | **Target deconvolution** | | | | | **Pathway deconvolution** | | |
| --- | --- | --- | --- | --- | --- | --- | --- | --- | --- |
|  |  | **Total connections** | **Connections targeting same molecule** | **% with same target** | **Connections targeting same protein family** | **% with same family** | **Total connections** | **Connections targeting same pathway** | **% with same pathway** |
| 0.74 | 1.0e-2 | 3668 | 268 | 7.3 | 335 | 9.1 | 3668 | 1048 | 28.6 |
| 0.80 | 7.9e-3 | 2005 | 177 | 8.8 | 222 | 11.1 | 2005 | 630 | 31.4 |
| 0.85 | 6.7e-3 | 1234 | 124 | 10 | 158 | 12.8 | 1234 | 417 | 33.8 |
| 0.90 | 5.7e-3 | 738 | 90 | 12.2 | 115 | 15.6 | 738 | 268 | 36.3 |
| 0.95 | 4.9e-3 | 486 | 72 | 14.8 | 92 | 18.9 | 486 | 201 | 41.4 |
| 1.00 | 4.3e-3 | 343 | 53 | 15.5 | 67 | 19.5 | 343 | 141 | 41.1 |
| 1.05 | 3.7e-3 | 237 | 44 | 18.6 | 54 | 22.8 | 237 | 102 | 43 |
| 1.10 | 3.3e-3 | 173 | 34 | 19.7 | 39 | 22.5 | 173 | 80 | 46.2 |
| 1.15 | 2.8e-3 | 118 | 22 | 18.6 | 26 | 22 | 118 | 55 | 46.6 |
| 1.20 | 2.4e-3 | 87 | 19 | 21.8 | 22 | 25.3 | 87 | 41 | 47.1 |
| 1.25 | 2.0e-3 | 51 | 13 | 25.5 | 13 | 25.5 | 51 | 28 | 54.9 |
| 1.30 | 1.6e-3 | 36 | 10 | 27.8 | 10 | 27.8 | 36 | 23 | 63.9 |
